# Deep representation learning of tissue metabolome and computed tomography annotates NSCLC classification and prognosis

**DOI:** 10.1038/s41698-024-00502-3

**Published:** 2024-02-03

**Authors:** Marc Boubnovski Martell, Kristofer Linton-Reid, Sumeet Hindocha, Mitchell Chen, Paula Moreno, Marina Álvarez‐Benito, Ángel Salvatierra, Richard Lee, Joram M. Posma, Marco A. Calzado, Eric O. Aboagye

**Affiliations:** 1 Imperial College London Hammersmith Campus, London, SW7 2AZ UK; 2 Early Diagnosis and Detection Centre, National Institute for Health and Care Research Biomedical Research Centre at the Royal Marsden and Institute of Cancer Research, London, SW3 6JJ UK; 3 Instituto Maimónides de Investigación Biomédica de Córdoba (IMIBIC), Córdoba, 14004 Spain; 4 Departamento de Cirugía Toráxica y Trasplante de Pulmón, Hospital Universitario Reina Sofía, Córdoba, 14014 Spain; 5 Unidad de Radiodiagnóstico y Cáncer de Mama, Hospital Universitario Reina Sofía, Córdoba, 14004 Spain; 6 National Heart and Lung Institute, Imperial College London, Guy Scadding Building, Dovehouse Street, London, SW3 6LY UK; 7 Departamento de Biología Celular, Fisiología e Inmunología, Universidad de Córdoba, Córdoba, 14014 Spain

**Keywords:** Non-small-cell lung cancer, Cancer imaging

## Abstract

The rich chemical information from tissue metabolomics provides a powerful means to elaborate tissue physiology or tumor characteristics at cellular and tumor microenvironment levels. However, the process of obtaining such information requires invasive biopsies, is costly, and can delay clinical patient management. Conversely, computed tomography (CT) is a clinical standard of care but does not intuitively harbor histological or prognostic information. Furthermore, the ability to embed metabolome information into CT to subsequently use the learned representation for classification or prognosis has yet to be described. This study develops a deep learning-based framework -- tissue-metabolomic-radiomic-CT (TMR-CT) by combining 48 paired CT images and tumor/normal tissue metabolite intensities to generate ten image embeddings to infer metabolite-derived representation from CT alone. In clinical NSCLC settings, we ascertain whether TMR-CT results in an enhanced feature generation model solving histology classification/prognosis tasks in an unseen international CT dataset of 742 patients. TMR-CT non-invasively determines histological classes - adenocarcinoma/squamous cell carcinoma with an F1-score = 0.78 and further asserts patients’ prognosis with a c-index = 0.72, surpassing the performance of radiomics models and deep learning on single modality CT feature extraction. Additionally, our work shows the potential to generate informative biology-inspired CT-led features to explore connections between hard-to-obtain tissue metabolic profiles and routine lesion-derived image data.

## Introduction

Numerous studies have developed imaging analysis pipelines to analyze and diagnose lung cancers. Tools such as radiomics have been helpful in extracting features from CT scans of lung cancer patients, followed by machine learning models to perform classification or prognosis tasks^1–3^. These features quantify the tumor’s spatial complexity, such as shape, size and intensity features and are becoming part of a routine investigation in the literature^3^. More recently, some studies have attempted to replace radiomic features with deep learning (DL) features extracted from convolutional neural networks (CNN) directly on lesions^4–7^. Of various CNN architectures, autoencoders are some of the most widely adopted and aim to find features that allow a model to reconstruct the original image in a different context^4,7,8^. In the context of diagnosing from CT scans, DL methods tend to outperform traditional radiomics feature extraction and selection methods^9,10^.

However, in practice, these features have limited clinical performance, e.g. classification C-index = 0.65, and the used framework precludes intuitive biological or clinical interpretability^11^. Current studies generate radiomic features and subsequently check if they associate with genes, metabolites, proteins and other biological factors, using for example, gene-set enrichment analysis^12–14^. In contrast, our present study develops a framework for generating features from images that have already learned specific biological representations of tissue metabolites.

A new and evolving field of computational biology combines two or more diverse modalities to improve the performance of each^15–17^. Gundersen and colleagues demonstrated the feasibility of developing multimodal pairings of pathology and genomic profiles to extract deep features from pathology images connected to the genomic profiles of the patient to obtain more explainability^18^. Once developed, the corollary of this learned representation approach, implies that one of the two modalities is sufficient to represent the other in the absence of both modalities being present^18^. Inspired by this approach, we investigate whether it is possible to generate deep features from hard-to-obtain tumor and normal tissue metabolome data, on the one hand, and the more routine CT scan image data on the other.

In characterizing tumors, the choice of metabolomics profiles as a benchmark is predicated on our recent work that expounds the use of tumor and adjacent tissue metabolome information in asserting the classification of histology subtypes, achieving an F1-score of 0.96, significantly outperforming most published models from imaging data in the field of lung cancer subtype classification or prognosis^19,20^. While the chemical information from tissue metabolomics is rich, the approach is not routinely used in patient management due to its invasiveness and analytical complexity^21^. Thus, we have developed a pipeline using an autoencoder to investigate, for the first time, whether the deep features of CT image reconstructions linked to chemical information from the metabolomics of patients will provide sensitive clinical information. These deep features extracted from the deep probabilistic canonical correlation analysis (DPCCA) model were named tissue metabolomic radiomic computed tomography (TMR-CT). The model aims to establish a connection between both data modalities.

**Fig. 1:**
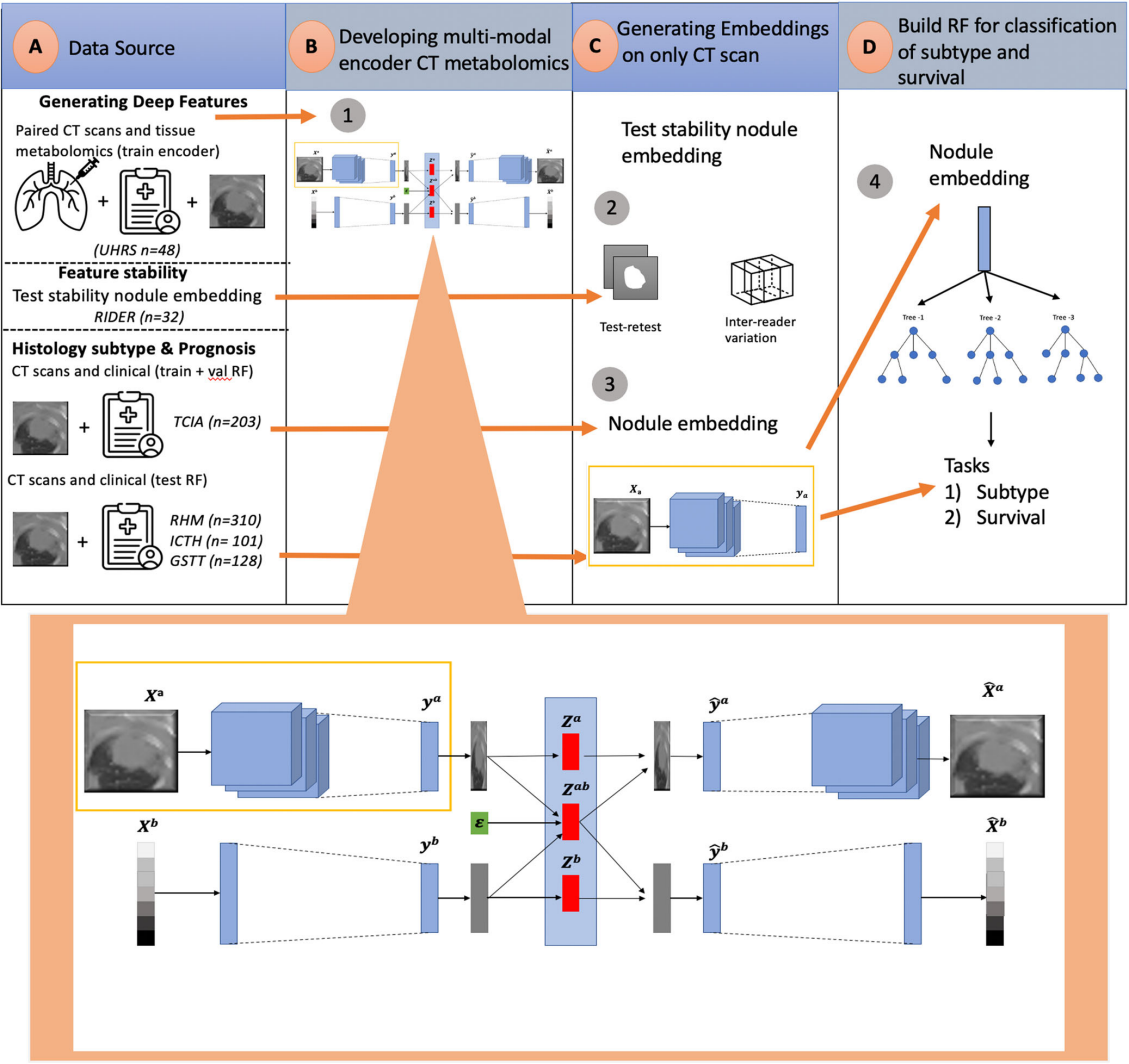
Study workflow. **a** Dataset collection for generating deep features, evaluating feature stability, histology subtype classification and prognosis. **b** The DPCCA model is used to find a shared latent space between the CT scans and metabolomics. An enlarged version of this model is shown, with the purple box highlighting the section responsible for generating TMR-CT features. In this model, *X*^*a*^, *X*^*b*^ is the original paired image and metabolomics; from these, we create *y*^*a*^*,y*^*b*^ image and metabolomics embeddings, respectively. The PCCA model then combines them into *z*^*a*^,*z*^*ab*^,*z*^*b*^ latent variables. The latent variables *z*^*a*^*,z*^*b*^ capture view-specific variation while *z*^*ab*^ captures the covariance. From the latent variables, we use a generative process of the model-sampling from the low dimension PCCA to reconstruct image and metabolomics embeddings ${\hat{y}}^{a},{\hat{y}}^{b}$. Each embedding ${\hat{y}}^{a},{\hat{y}}^{b}$ is then decoded to produce the ${\hat{X}}^{a},{\hat{X}}^{b}$ using view-specific decoders. During implementation, when we only have the CT image, we extract the learned representation *y*^*a*^, which we have defined as the TMR-CT features. **c** Convolutional neural networks within the purple box were used to generate TMR-CT features on external datasets and to test the stability of the features using the RIDER dataset. **d** TMR-CT features are utilized for histology subtype classification, with random forest (RF)-based approaches displayed, as they exhibited superior performance in both tasks.

The model comprises a two-stage neural network and PCCA. The neural network part first finds separate embeddings for each data modality (CT scan, metabolites). These embeddings are subsequently combined to maximize correlation and minimize reconstruction loss for both modalities^18^. The benefit of this structure is two-fold. First, the deep features captured for each data view maximize the shared variation. Second, the generative structure of the model allows cross-data modality imputation. This is particularly important given the difficulty in obtaining the paired datasets from both modalities^19^. We explore the aforementioned benefit in this manuscript by utilizing the embeddings derived from CT scans for histology subtype classification and prognosis of non-small cell lung cancer (NSCLC) patient data. Our approach achieves an enhanced feature generation model in both tasks while also providing valuable biological insights. We accomplish this by employing TMR-CT, which encompasses reconstructed representations of tissue metabolite types and intensities. This work demonstrates the potential for the method to enhance the practitioner’s – radiologist’s, respiratory physician’s, or oncologist’s – ability to determine histology subtype classification, as well as prognosis, using algorithms derived from the current work, as illustrated in Fig. 1.

## Results

### Datasets

The datasets in our study can be split into three parts, as seen in Fig. 1 (data source): developing deep features, testing feature stability, histology classification and prognosis.

The dataset from 48 patients with both tissue and CT scans used to develop TMR-CT was obtained from the University Hospital Reina Sofia (UHRS), Spain. The research study was conducted in accordance with the Helsinki Declaration and was approved by the Cordoba Clinical Research Ethics Committee, all patients provided a signed written informed consent for participation in the study. Paired CT and tissue, obtained from both the tumor and non-tumor adjacent tissues, were collected from patients with NSCLC. Tissue samples were stored by the Andalusian Health Services Biobank, and the metabolomic profiling was performed under contract by Metabolon^19^. The patients did not receive any radiation or chemotherapy treatments before surgical resection, and the clinicopathological information was obtained prospectively and shown in Table 1. All tissue data were processed as previously reported^19^. CT scans were segmented by a board-certified clinical radiologist (MC).

**Table 1:** Patient demographics in the dataset from University Hospital Reina Sofia, Spain, with joint CT and metabolomics used for developing TMR-CT.

		(*n* = 48)		
	Characteristics	AC^†^	SCC^†^	*p*-value
	Patients	22	26	
	Age	61.7 (±16.9)	70.3 (±7.3)	0.27
Gender	Male	16	26	0.006
	Female	6	0	
TNM8 Overall stage	1	18	17	0.53
	2	3	7	
	3	1	2	

^†^*AC* adenocarcinoma of the lung, *SCC* squamous cell carcinoma of the lung.

Metabolite analyses were performed as previously described^19^. Samples were extracted by an aqueous methanol extraction process and analyzed with ultra‐performance liquid chromatography/tandem mass spectrometry (UPLC/MS/MS; positive mode), UPLC/MS/MS (negative mode), and GC/MS by Metabolon. Tissue metabolites were identified by comparison with library entries of purified standards or recurrent unknown entities. Based on the literature and KEGG/HMDB databases, metabolites were annotated to one of eight ‘super pathways’ corresponding to their general metabolic processes (amino acid, lipid, carbohydrate, nucleotide, peptide, energy, cofactors and vitamins, and xenobiotics), and to one of 73 ‘sub pathways’ representing more specific metabolic pathways or biochemical subclasses; in the aggregate, 851 metabolites were identified through this approach for both lung adenocarcinoma (AC) and squamous cell carcinoma (SCC) subtypes, and normal lung tissues^19^.

To test the stability of the TMR-CT features, we used the open-sourced RIDER dataset consisting of 32 patients with NSCLC who underwent two sequential chest CT scans within 15 mins, employing the same imaging protocols^22^. In this study, three radiologists measured the two greatest diameters of each lesion on both scans obtaining highly reproducible measurements, all with concordance correlation coefficients (CCC) greater than 0.96. Thus, this dataset has been shown to be useful in determining the reproducibility of deep learning features for NSCLC^23^.

To test how useful TMR-CT is for histology classification and prognosis prediction, we used four different datasets summarized in Table 2. To train our models, we used the open-source TCIA (The Cancer Imaging Archive), from which we selected 203 patients diagnosed with either AC or SCC^24^. The TCIA was split into 120 for training and validation and 83 for external validation. Then, to evaluate how well the developed model generalize to new NSCLC datasets, we used three geographically distinct datasets from the OCTAPUS-AI study (ClinicalTrials.gov identifier: NCT04721444) as external test sets (GSTT, Imperial and RMH); OCTAPUS-AI represents a study from multiple UK cancer centers (Guy’s and St Thomas’ NHS Foundation Trust, Imperial College Healthcare NHS Trust and the Royal Marsden NHS Foundation Trust respectively) collected for the explicit purpose of developing robust predictive lung cancer algorithms^25^. As the data were deidentified, patient consent was not required as per the respective Health Research Authority and Research Ethics Committee approvals.

**Table 2:** Patient demographics and treatment variables in the three external datasets used for histology classification and prognosis (for age and radiotherapy dosage, we show the median value together with the IQR in brackets).

		Train and Validation		External test						
		TCIA (*n* = 203)		GSTT (*n* = 128)		ICHT (*n* = 101)		RMH (*n* = 310)		*p*-value
	Characteristic	AC	SCC	AC	SCC	AC	SCC	AC	SCC	<0.001
	Patients	152	51	67	61	49	52	189	121	
	Age (IQR)	68 (±15)	71 (±14)	70 (±15)	73 (±11)	71 (±14)	72 (±11)	74 (±17)	76 (±12)	
Gender	Male	32	112	36	39	27	33	83	82	<0.001
	Female	19	40	31	22	22	19	106	39	
CT type	Contrast			29	23	29	31	55	42	
	non-contrast			38	37	20	21	134	79	
Dosage	Biologically effective dosage, Gy			77 (±39)	77 (±35)	70 (±9)	70 (±9)	77 (±39)	72 (±23)	
End result	Survival days	583	492	864	760	895	867.7	834	694	0.35
	Recorded deaths	45	139	32	40	26	42	104	79	0.22
Treatment	Conventional RT only			10	19	22	29	31	31	
	SBRT			29	17	0	0	90	31	
	Sequential chemoRT			10	11	9	9	37	37	
	Concurrent chemoRT			18	14	18	14	31	18	
TNM8 Overall Stage	1			32	23	10	8	89	36	
	2			10	9	13	12	22	22	
	3			25	29	26	32	78	63	
Slice thickness	2			0	0	0	0	172	114	
	2.5			67	61	0	0	17	7	
	3			0	0	49	52	0	0	

### Overview of metabolic profiles for NSCLC

**Fig. 2:**
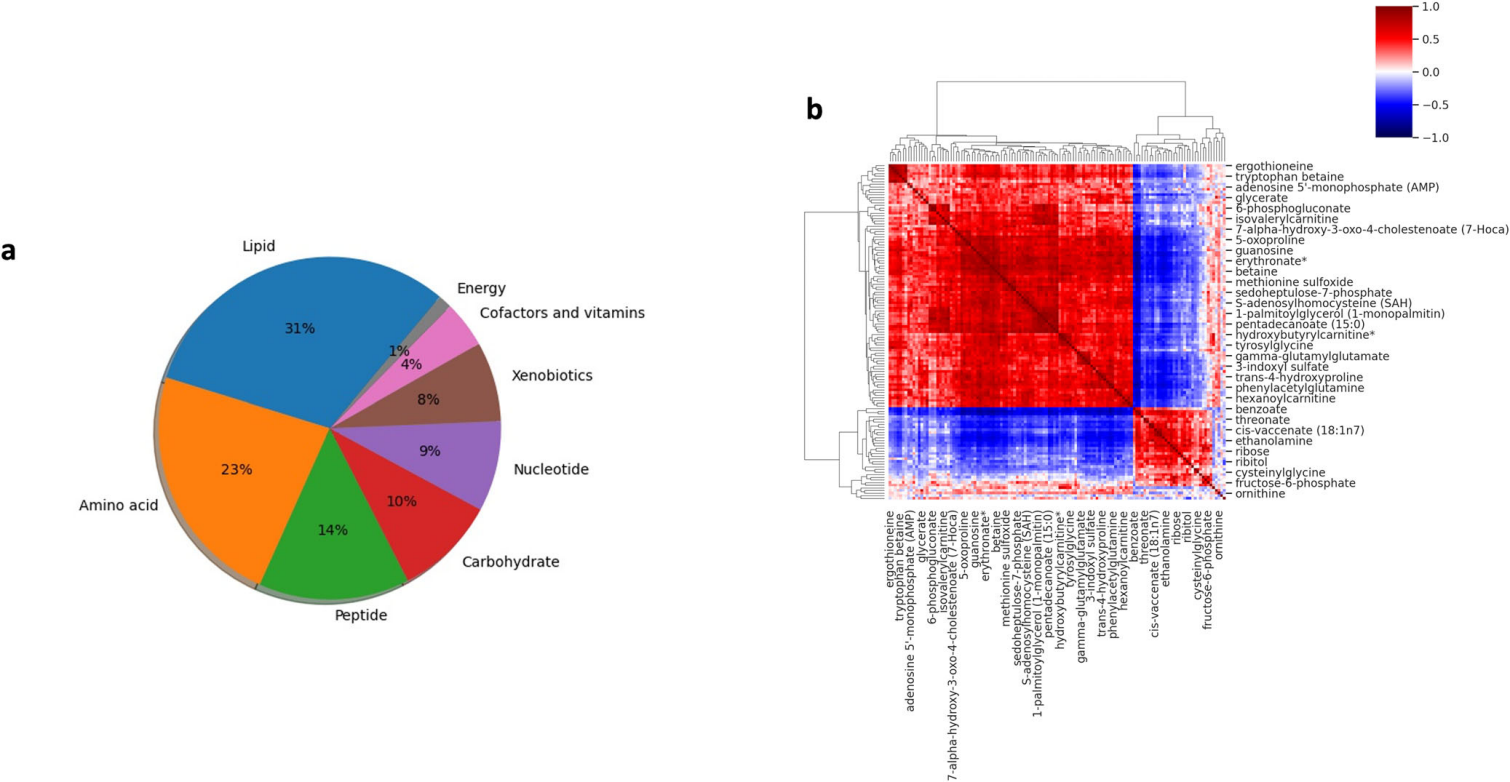
Representation of congruent metabolomic information across all patients. **a** Distribution of metabolites super pathway, which were present in all patient’s data used in the current study. **b** Pearson correlation heat map between tumor metabolomics for all patients.

With many more metabolomic features than patients, we first filtered the metabolomics by only including those profiled in all 48 patients for both tumor and non-tumor adjacent tissue from the UHRS hospital; this reduced the number of metabolites to 174. The super pathway of these features is summarized in Fig. 2a, and we observed a high degree of positive and negative correlation between several of the features from the tumor tissue samples, as shown in Fig. 2b.

Despite the large number of metabolomic features compared to our sample size, we did not need to perform feature reduction, as principal component analysis (PCA) is known to be robust to correlated features^26^. Furthermore, we reasoned that when incorporating the metabolomics feature into the DPCCA model, we would perform data augmentation as specified in “Pre-processing data for training and testing DPCCA” to mitigate overfitting.

We were unable to use permutation importance to identify the most important metabolomic features due to feature collinearity phenomenon; permuting any single feature would have little effect on the random forest (RF) performance. As an alternative, we performed hierarchical clustering on the Pearson rank-order correlation and chose a single feature from every cluster, as suggested by Rosato and co-workers in a systems biology-enhanced analytical framework for metabolomics data^27^. This approach allowed us to reduce the number of features to six metabolomics (1,5-anhydroglutocitol (1,5-AG), 1-arachidonoylglycerophosphoethanol-amine*, 1-stearoylcerol (1-monostearin), 3-hydroxybutyrate(BHBA), 3-phosphogylcerate and alanine) while still maintaining the same performance (F1-score of 1) in discriminating AC from SCC tissue.

### Ordinarily metabolites discriminate histology subtypes but are unconnected to radiomic features

**Fig. 3:**
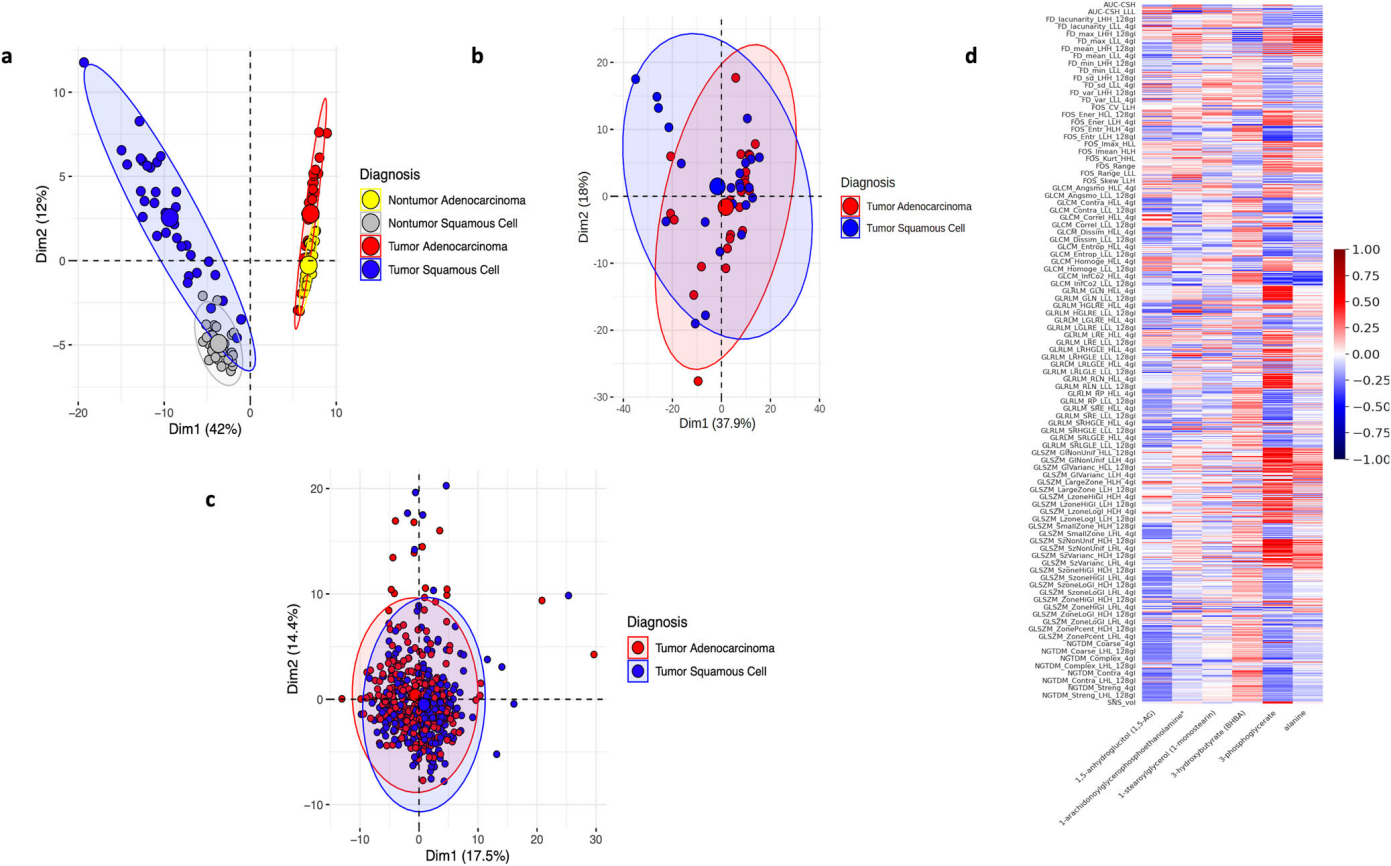
investigating overlap of metabolomics and radiomics data structures. **a** Two-dimensional PCA of metabolomic features from UHRS dataset. **b** Two-dimensional PCA of radiomic features from UHRS dataset. **c** Two-dimensional PCA of radiomic features from a larger TCIA dataset. **d** Pearson correlation heatmap between CT radiomic features and six metabolomic features important for classification of histology subtypes from UHRS dataset.

We investigated the data structure of the metabolomic profiles obtained from 48 tumor and non-tumor tissue samples in relation to the radiomic features from CT scans. For each CT scan, we extracted 438 radiomic features using the TextLab 2.0 software related to shape, size, intensity, and wavelet decomposition^1^. After pairing metabolomic and radiomic features for the 48 samples, we compared the predictive power and connection between both modalities. To determine the predictive power of both modalities, we examined 2-dimensional PCA with all variables. We found that the metabolomics provided more informative predictions of tissue subtypes compared to CT radiomics Fig. 3a–c. Determining the most important metabolomic features, proved challenging as described in “Overview of Metabolic Profiles for NSCLC” and we used hierarchical clustering based on the Pearson rank-order correlation.

Due to the large number of radiomic features, it is common practice to perform a feature reduction step prior to model building. While there is no established set way, various task-dependent strategies have been proposed^28^. For comparison, we only retained features with an intra-class-correlation coefficient (ICC) greater than 0.75, resulting in 438 features^29^.

To investigate the connection between the two sets of features within the tumor metabolomics and imaging datasets, we conducted a Pearson correlation analysis between the radiomics and the top ten metabolomics features. A heatmap of the data is shown in Fig. 3d. With a maximum absolute correlation coefficient of 0.45 and a mean correlation of 0.02, the results suggested a weak correlation between the two data modalities and techniques. Consequently, traditional canonical correlation analysis might be inappropriate.

### The DPCCA model can be trained on CT scans and metabolomics to define TMR-CT

**Fig. 4:**
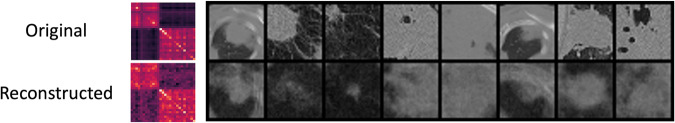
Reconstructing the metabolite-inspired CT scan. To test the quality of our latent space model developed by DPCCA, we examined CT and metabolomics reconstruction. The images above were obtained from the test data for different patients (Top row). The original metabolomics expression covariance matrix and random CT slices from test data and (Bottom row) the reconstruction of the CT image of unseen test dataset when both the original image and metabolites are provided as inputs to the model.

DPCCA was trained to expound the shared latent space between metabolomics and CT scan data. To evaluate the performance of DPCCA, we assessed the following. Firstly, we investigated if our model could reconstruct both modalities from the shared latent space. Thus, we examined the reconstructed CT slices and the metabolomic covariance matrix on the held-out test dataset. As seen in Fig. 4, DPCCA successfully reconstructed both views. As enshrined in a similar inference framework by Gundersen and co-workers for gene expression and latent pathology space^18^, we wanted to verify that our end-to-end model, composed of a neural network and DPCCA, makes use of both components. To test this, we computed the expected complete negative log-likelihood on the held-out dataset. A limitation of our work relates to the restricted training sample size, which could lead to overfitting when using DPCCA for training. As shown in Supplementary Fig. 1, the loss function of the validation set decreased during training for two modalities similar to those observed in a previously published study that reported DPCCA. As a baseline, we compared its performance to how well the image component of the DPCCA image autoencoder can reconstruct the image modality. As expected, and noted in an earlier study, the single modality is faster to train and has smaller reconstruction loss than the DPCCA which aims to reconstruct both views^18^.

Tumor size, CT scan thickness, and manufacturer can impact the outcomes. Principal component analysis (explained variance) outputs of TMR-CT, to assess congruence of data from TNM8 stage and CT scan thickness are illustrated in Supplementary Figs. 2 and 3, and show that these variables have no impact on the TMR-CT. We also examined the correlation between tumor size and the TMR-CT features and found that the maximum absolute correlation was weak (0.32, *p* = 0.03).

**Fig. 5:**
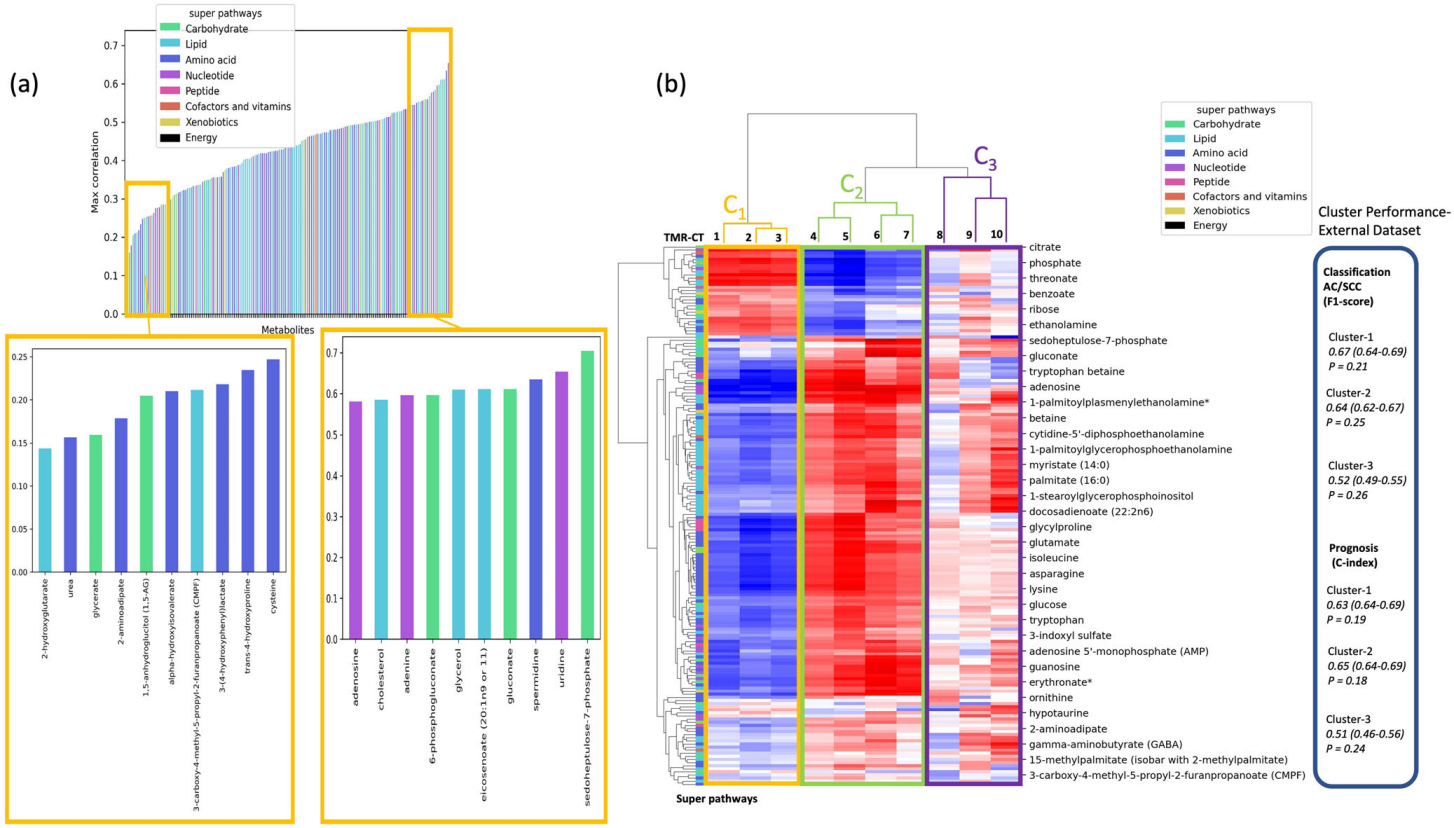
A description of metabolites emphasized by TMR-CT. **a** Correlation of performance of the TMR-CT model developed by DPCCA, the bar plot shows the highest absolute correlation between metabolites and the TMR-CT features; we expand the top ten least correlated metabolites and the top ten most correlated metabolites in the yellow boxes. **b** Unsupervised hierarchical clustering of the TMR-CT features with the metabolomic profile from nodule UHRS identified three distinct subgroups. The blue rectangle contains a summary of the performance of the distinct cluster for classification and prognosis. The pentose phosphate metabolite sedoheptulose-7-phosphate, an important source for ribonucleotides and reduced nicotinamide adenine dinucleotide phosphate (NADPH), adenosine, reported in our previous lung metabolomics publication to be high in tumor tissue are emphasized in both the highly correlated metabolite set (**a**) and cluster 2 (**b**). Other metabolites such as asparagine, an important regulator of cancer cell amino acid homeostasis, anabolic metabolism and proliferation are emphasized only by the clustering approach^19,45,46^.

To gain a good understanding of metabolomic features that are the focus of the study, we plotted the maximum absolute correlation between the metabolomic features and the ten TMR-CTs in Fig. 5a. The two metabolites with the lowest correlation are 2-hydroxyglutarate and urea, with a maximum correlation of 0.14 and 0.16, respectively. The two metabolites with the highest correlation are sedoheptulose-7-phosphate and uridine, with a correlation of 0.72 and 0.64, respectively.

It is important to note that the focus of the DPCCA model was to find a shared latent space and then reconstruct the learned representation; thus, the ‘metabolomic features’ that the model focuses on are not necessarily those with the greatest classification or prognosis power, but rather those that the model can use in representing the CT-images. To interpret the influence of metabolomics on the shared embeddings, we plotted an unsupervised hierarchical clustering of the TMR-CT correlations in Fig. 5b, which shows the presence of three clusters with relevant metabolite super pathway information on the test dataset of the UHRS.

We see how the three clusters correlate very differently to the metabolomic features, showing that they complement themselves and model different parts of the metabolomic features. This shows that the DPCCA has achieved the goal of generating different features that capture the entire metabolomic profile by through correlation, though the exact biological pathways used in the correlation is not intuitive. A small subset of metabolites at the bottom of the map has a low correlation with all three clusters, suggesting that the DPCCA model could not correctly identify them in the CT image. The performance of each cluster on classification and prognosis appears unique; clusters C_1_ and C_2_, while showing opposite correlation values to most metabolites demonstrated similar performance in both histology classification and prognosis, which C_3_ performed less well in both tasks.

### Reliability and reproducibility of TMR-CT

To assess the stability of our encoder algorithm, we tested it in a test-retest context using the publicly available dataset, RIDER, consisting of 32 patients with lung cancer. Each patient underwent two chest CT thorax scans (within 15 min apart) using the same imaging protocol^22^. We evaluated the stability of the encoder by examining the TMR-CT features between the test and retest scans. Our results demonstrated a high level of stability with an ICC of 0.86 for TMR-CT showing that our model had been well-regularized.

To account for inter-reader stability, we adopted an approach of relocating the input seed points to the center of the tumor. This aimed to simulate various radiologists annotating the tumor, which would cause variability between them. In this case, we showed a high correlation with a Spearman’s rank-order correlation of 0.85 between the TMR-CT, showing strong inter-reader stability.

### Exploiting TMR-CT features from DPCCA for classification and prognosis of CT scans without metabolomic profiles

**Fig. 6:**
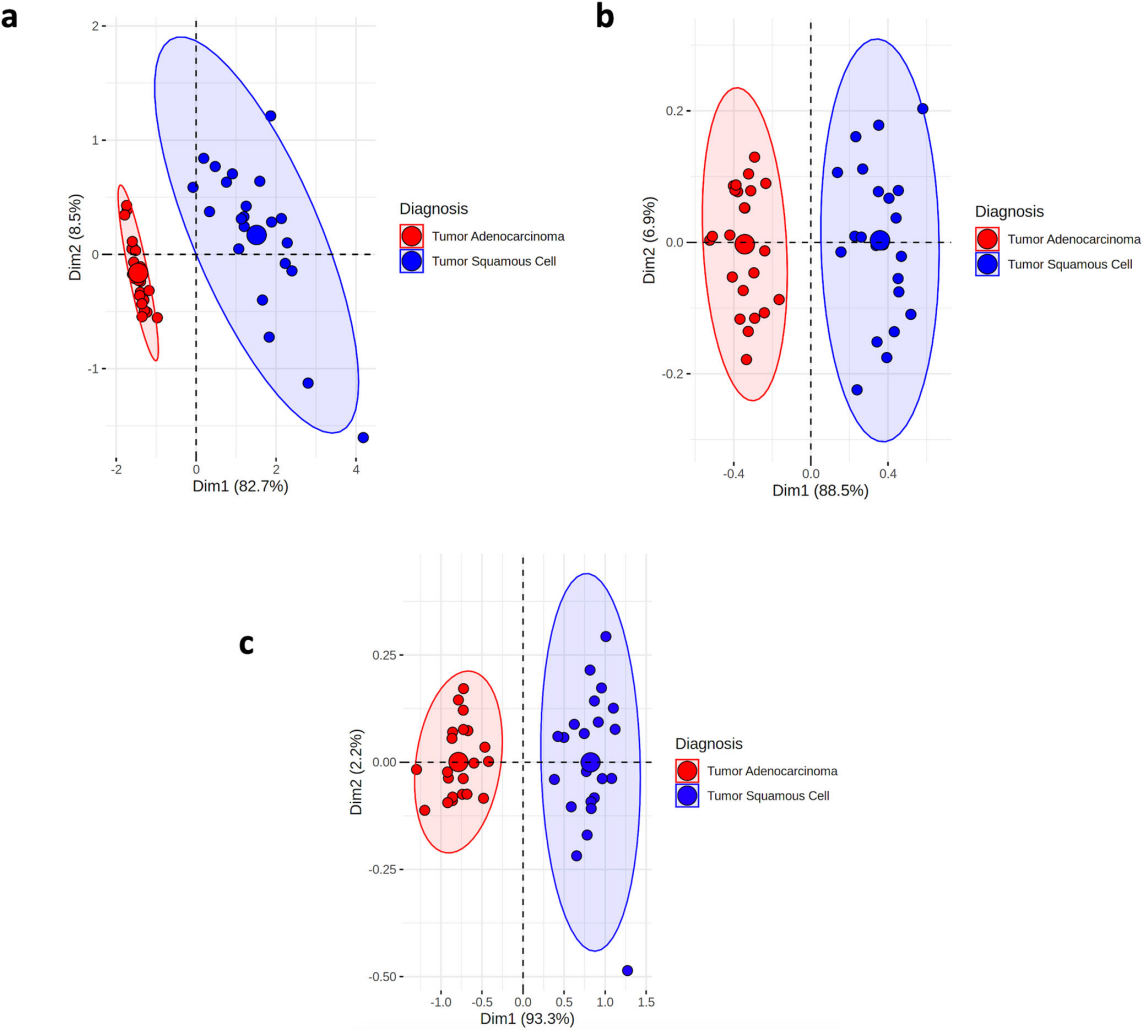
Information content of view-specific and shared embedding space. **a** TMR-CT (image embeddings space *y*^*a*^) (**b**) metabolomic embeddings *y*^*b*^ and (**c**) image and metabolomic shared latent space *z*^*ab*^.

**Fig. 7:**
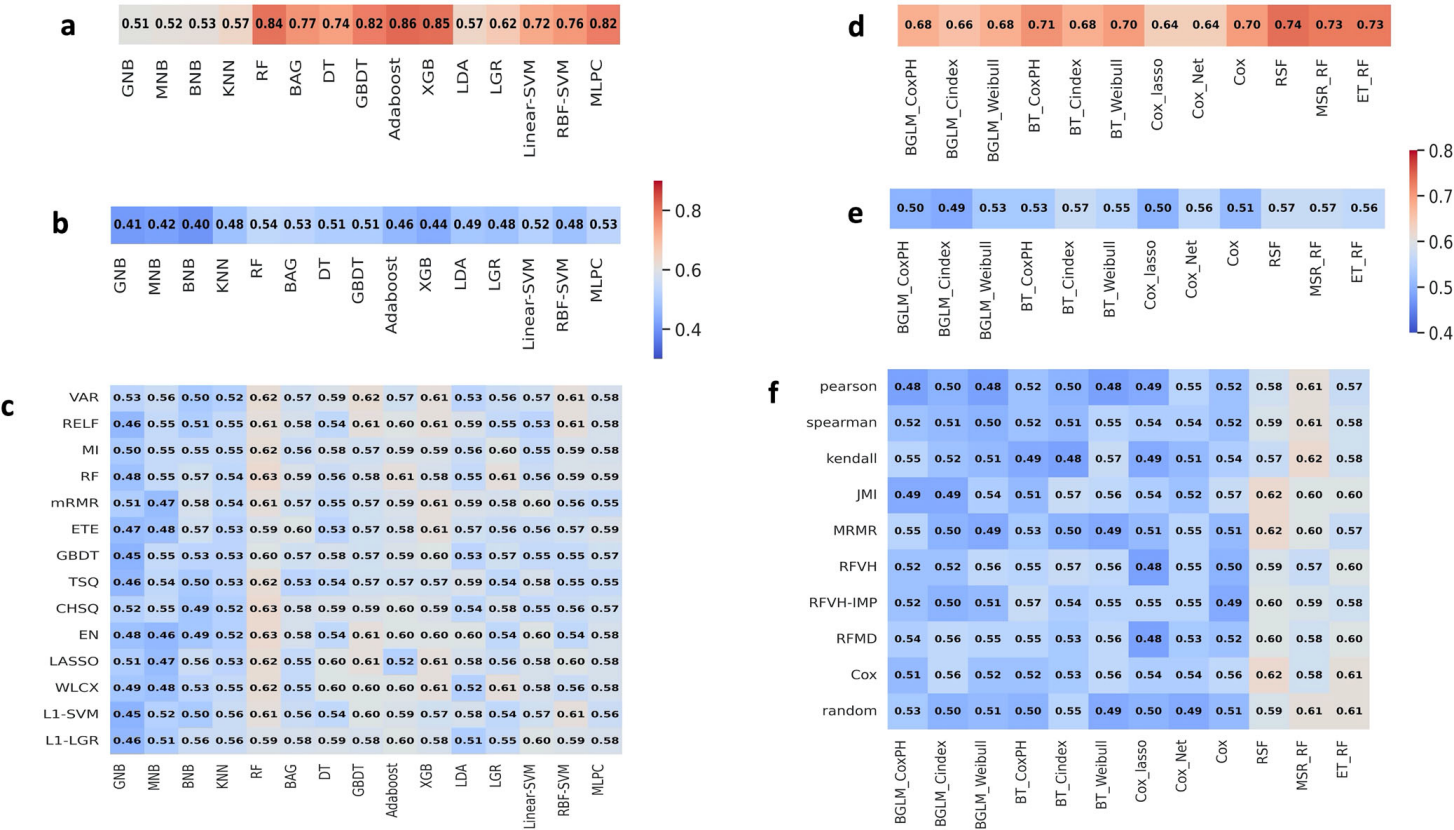
Performance of different models developed for classification or prognosis. **a** F1-score for classification of AC and SCC using TMR-CT for different classification models. **b** F1-score for classification of AC and SCC using CT_emb for different classification models. **c** F1-score for classification of AC and SCC using radiomic features, with the *x*-axis being the predictive models and the *y*-axis corresponding to feature selection techniques. **d** C-index for a prognosis for different models using TMR-CT. **e** C-index for a prognosis for different models using CT_emb. **f** C-index using radiomic features, with the *x*-axis being the prognosis models and the *y*-axis corresponding to feature selection techniques.

We aimed to determine if our latent variables captured meaningful, held-out biological information such as histology subtype and overall survival (OS). To have an overview of the information captured in the shared and view specific embedding space from the DPCCA model we plotted the PCA of the TMR_CT features in Fig. 6. Similarly, we plotted the PCA for the CT_emb features in Supplementary Fig. 4 and observed how the image embeddings are less informative compared to Fig. 6. However, to get a better understanding of how these features can be useful in downstream tasks, we trained separate models and feature selection were tuned on TCIA data as seen in Fig. 7, to select the best model for each task. In the case of the radiomics features we also selected the best feature selection technique. With many radiomic features (438), the feature selection technique is particularly important to optimize the model and make it directly comparable to the TMR-CT and CT_emb features which both have 10 features respectively. After performing feature reduction using the techniques showed in Fig. 7 the number of remaining features ranged from four to thirteen depending on the feature reduction method. More specifically the best radiomic models for histology classification and prognosis used six and eight features, respectively, thus being the same order of magnitude as TMR-CT features. The features used in the best feature selection model combination are the following: six features for histology classification (FD_max_LLH_25HUgl, GLRLM_RP_LHL_25HUgl, GLCM_Entrop_HLH_25HUgl, GLCM_AutoCorrel_LLL_25HUgl, GLCM_invVar_LLL_25HUgl and FOS_RMS_LLL). The best prognosis model uses eight (SNS_max3d, FD_max_LLL_25HUgl, FD_max_LLH_25HUgl, GLCM_invVar_25HUgl, FOS_RMS_LLL, GLCM_invVar_LLL_25HUgl, GLCM_IDN_LLH_25HUgl and NGTDM_Coarse_LLL_25HUgl). Where SNS = size and shape features, FD = fractal dimensions, GLCM = Grey-level co-occurrence matrix, GLSZM = Grey-level size zone matrix, FOS = first order statistics, GLRLM = Grey-Level Run Length Matrix, NGTDM = Neighborhood grey- tone difference matrix, GLCM = Grey-level co-occurrence matrix).

From Fig. 7, it is evident how RF using TMR-CT features significantly outperforms the CT_emb and traditional radiomics features extracted using TextLab 2.0 for histology classification and prognosis without performing any feature selection. This finding is important as it shows that the quality of the TMR-CT features is sufficiently high. Thus, no feature selection is required. This can be further seen in Tables 3 and 4 where we chose the best performing feature selection and model, respectively, from Fig. 7 to report the results on the four external datasets and the ROC curves are plotted in Supplementary Fig. 5.

**Table 3:** F1-score of RF for classification of AC and SCC reported using the best feature selection and machine learning model ± standard error.

	TCIA ext val		RMH (*n* = 320)		GSTT (*n* = 128)		ICHT (*n* = 101)	
Features	F1-score	*P*-value	F1-score	*P*-value	F1-score	*P*-value	F1-score	*P*-value
Radiomics	0.63 ± 0.02	0.15	0.58 ± 0.04	0.32	0.59 ± 0.03	0.19	0.57 ± 0.02	0.20
CT_emb	0.60 ± 0.04	0.18	0.54 ± 0.05	0.33	0.53 ± 0.06	0.17	0.56 ± 0.05	0.30
TMR-CT	0.84 ± 0.03	0.09	0.78 ± 0.02	0.21	0.77 ± 0.03	0.16	0.79 ± 0.03	0.23

TCIA, RMH, GSTT and ICHT are The Cancer Imaging Archive, Royal Marsden Hospital (UK), Guy’s and St Thomas’ Hospital (UK) and Imperial College Healthcare Trust (UK), respectively.

**Table 4:** C-index of Random Survival Forest for prognosis of NSCLC reported using the best feature selection and machine learning model ± standard error.

	TCIA ext val		RMH (*n* = 320)		GSTT (*n* = 128)		ICHT (*n* = 101)	
Features	C-index	*P*-value	C-index	*P*-value	C-index	*P*-value	C-index	*P*-value
Radiomics	0.62 ± 0.04	0.05	0.58 ± 0.05	0.04	0.61 ± 0.04	0.01	0.59 ± 0.06	0.001
CT_emb	0.57 ± 0.06	0.42	0.53 ± 0.07	0.89	0.52 ± 0.06	0.96	0.54 ± 0.06	0.54
TMR-CT	0.74 ± 0.03	0.30	0.72 ± 0.04	0.73	0.71 ± 0.05	0.84	0.71 ± 0.04	0.34
Radiomics + clinical	0.64 ± 0.05	0.20	0.59 ± 0.06	0.06	0.62 ± 0.06	0.92	0.58 ± 0.07	0.03
CT_emb + clinical	0.59 ± 0.05	0.45	0.55 ± 0.06	0.76	0.54 ± 0.07	0.92	0.55 ± 0.07	0.63
TMR-CT + clinical	0.78 ± 0.06	0.05	0.73 ± 0.06	0.3	0.71 ± 0.04	0.03	0.71 ± 0.05	0.12

TCIA, RMH, GSTT and ICHT are The Cancer Imaging Archive, Royal Marsden Hospital (UK), Guy’s and St Thomas’ Hospital (UK) and Imperial College Healthcare Trust (UK), respectively.

The prognosis was determined using the models alone or in a multivariable model with clinical features.

**Fig. 8:**
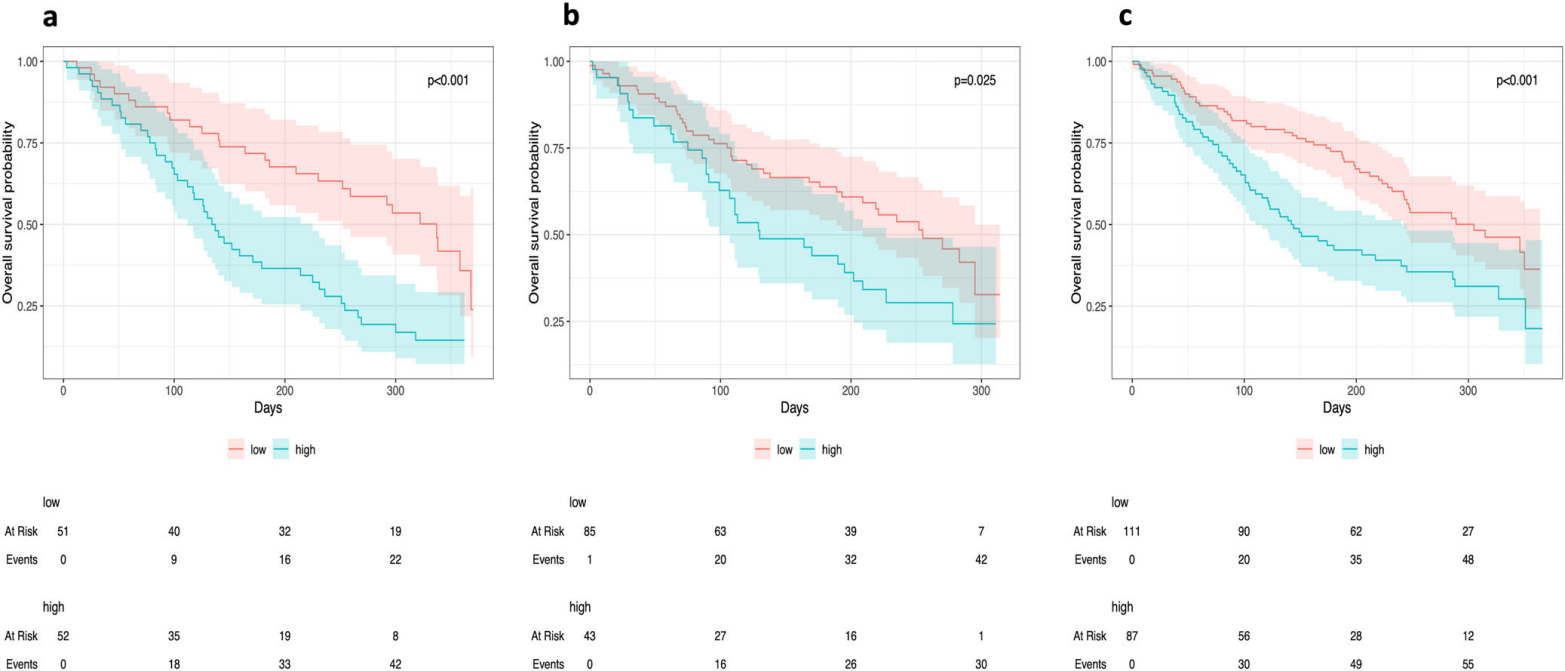
Estimating percent survival over time - Kaplan Meier plots - for dicotomised low and high-risk groups. Dichotomized predicted probabilities using k-means clustering of the RSF with TMR-CT on the external validation dataset: (**a**) ICHT, (**b**) GSTT and (**c**) RMH (*P*-values are from log-rank tests. Plots demonstrate good separation between high and low risk groups with log-rank tests confirming the statistical significance of 5% in the GSTT and 1% in ICHT and RMH.

The three external test sets of Kaplan Meier curves are shown in Fig. 8 and demonstrate good separation between high and low-risk groups with log-rank tests confirming a statistically significant difference a 5% level in the GSTT and 1% for the ICHT and RMH.

To understand the importance of different features in our best prognosis model noted in Table 4, we reported the hazard ratio that our model calculated together with the *P*-value for the log-rank test for each feature modality in Table 5. We observe in a multivariable model that TMR-CT exhibited the highest hazard values in both the validation and external test datasets showing the high importance of TMR-CT for prognosis. This finding is notably more significant than clinical features, including age, gender, N-stage and gross tumor volume (GTV).

**Table 5:** Hazard Ratio (HR) and *P*-values for Permutation-Variable Importance Random Forest – Random Survival Forest (PVIRF-RSF) Model combining Clinical and TMR-CT ± standard error.

	TCIA ext val		GSTT		ICHT		RMH	
Features	HR (95% CI)	*P*-value	HR (95% CI)	*P*-value	HR (95% CI)	*P*-value	HR (95% CI)	*P*-value
TMR-CT	38.0 (30.3–45.7)	>0.00001	17.1 (8.7–25.5)	0.04	15.9 (10.2–23.7)	>0.01	24.4 (17.3–31.5)	>0.001
Age	1.24 (0.83–1.65)	0.3	2.14 (1.63–2.65)	0.73	1.04 (0.66–1.41)	0.84	1.15 (0.92–1.38)	0.34
Gender	1.53 (1.12–1.94)	0.2	3.20 (2.91–3.49)	0.06	1.05 (0.58–1.53)	0.92	3.02 (2.21–3.83)	0.03
N-Stage	-	-	1.16 (0.89–1.43)	0.12	2.33 (1.76–2.90)	0.03	4.30 (2.93–5.67)	0.12
GTV	-	-	1.04 (0.52–1.56)	0.65	1.00 (0.46–1.64)	0.5	1.15 (0.71–1.59)	1.14

## Discussion

In this study, we have shown that a deep learning framework - DPCCA - can model a connection between CT scans of lung nodules and their tissue metabolomics profiles against the premise that certain metabolites and/or their intensities, representing tumor growth and/or tumor microenvironment factors maximally co-vary together, linearly or non-linearly, with CT image features. Furthermore, we have shown the usefulness of such models, embodied within TMR-CT, for histology subtype classification and prognosis of NSCLC patients non-invasively, thus asserting clinical relevance. Notably, the DPCCA-generated learned representations could be used for downstream classification or prognosis tasks even when we only have CT scans available. Such metabolomic profile-correlated features are more interpretable biologically. This methodology would be useful in guiding treatment decisions, particularly in the context of patients that are unfit for biopsy.

The generated metabolite pairs are not inherently intuitive. For example, the most important metabolites (in our DPCCA model) differ from our previously published top metabolites from metabolomics-only analysis, in the study by Moreno et al. (2018). One of the main reasons for this is that, in the metabolomics-only study, the top metabolites are chosen as those most discriminative between tumor and non-tumor cases for AC and SCC separately, obtaining 20 different metabolites^19^. In the current study, however, the top ten metabolites chosen are those most correlated with our TMR-CT, whose purpose is to reconstruct the two data modalities as a composite phenotype of both the CT features of the lesion and the metabolomics profile. Regardless, the most correlated metabolites appear to regulate cell growth and membrane activity through glycolysis, pentose-phosphate, DNA synthesis and fatty acid metabolism.

A fundamental point of consideration in this research is whether optimization of CT features, detached from metabolomics (using a radiomics pipeline; current state of the art in clinical setting) compares favourably with TMR-CT (generated by the DPPCA pipeline). Thus, we compared feature optimization employing a matrix of combinatorial feature reduction and machine learning model for prediction, detached from metabolomics information, with the DPPCA pipeline informed by tissue metabolomics. When comparing the two machine learning pipelines - TMR-CT versus radiomics optimised feature combinations - we observed that TMR-CT performed significantly better in two clinically distinct tasks: histology classification and prognosis. This suggests that TMR-CT optimized vector contains more relevant features than radiomics vectors, even though there are significantly fewer features in the former. This difference was observed regardless of whether TMR-CT was used alone or combined with patient information, meaning that TMR-CT exhibits overall superior performance compared to radiomic features for NSCLC histology classification and prognosis determination.

To our knowledge, over 16 different models have attempted to integrate multiomics using deep learning to gain a better understanding of the complex biological process of cancer^17^. However, most of these models aim to fuse multiomics of the same modality, making it a significantly easier process relying on the availability of both data modalities during the test time. Various studies have successfully integrated CT features with other biomarkers for lung cancer diagnosis and prognosis; however, a direct comparison is challenging due to differences in the datasets^30,31^. The said methods only work when both modalities are present^32^. Thus, the benefit of using DPCCA over other models is that it can be applied during test time even without information about the tissue metabolomic profiles (with only the CT available). We show that TMR-CT derived from DPCCA was superior to conventional radiomics for histology classification and prognosis in patients who only had the CT scan available.

Our current study exhibits a number of limitations. First, a potential limitation of the study is the sample size used for training. Of note, however, the DPCCA is particularly attractive for medical applications with a small sample size but a large feature space, as it explicitly models uncertainty^18^. In that study, DPCCA utilized two inputs: gene expression (18,659 features) and 2D tissue histology images of size (128 × 128) with three colour channels, so a total of 49,152 features. In our study, we used the 174 metabolomic features that were consistently present in all patients and CT images of size 32 × 32 × 32 with one colour channel and a total of 32,768 features. Although our dataset was slightly smaller with fewer features, this was compensated through data augmentations techniques, ultimately enabling our model to perform efficiently. Secondly, regardless of the aim of the approach is to permit future use of the more routine method (in this case CT), it is clear that we only validated the performance of the TMR-CT on external CT datasets but not metabolomics. In theory, one could inversely predict the values of metabolomics counterpart from CT data. This generative aspect of the model could be investigated when independent test tissue metabolomics data become available Future analysis on this independent cohort with paired CT and metabolomics data would be required to validate the stability of the correlations identified in our study. Lastly, a wider range of histology could have been used. In our study, we only examined patients with AC and SCC, but our technique could easily be extended to incorporate other lung cancer histology with minimal adaptation when those data become available.

In the future, two primary directions could be explored by researchers. The first is to validate the TMR-CT features on an external dataset of paired CT scans and metabolomics features. The second is to increase the number of patients in the paired CT and metabolomics dataset to contain a larger number of patients that are more representative of the wider population by including small cell lung cancer patients. Unfortunately, such datasets don’t currently exist, so we could not incorporate these ideas into our study. Nonetheless, by showcasing the efficacy of a niche algorithm in a specific context, we establish a foundation for future studies that aim to extend its performance and validation to diverse settings. By conducting a prospective study that combines TMR-CT, radiomics, and body fluid metabolomic analysis, it may be possible to improve prognostic capabilities when tissue metabolomics is unavailable. This is particularly relevant for patients who are deemed unsuitable for surgery or face obstacles in accessing tumor material for histology classification and prognostication prior to making a decision about surgery.

Our study investigates the feasibility of using deep learning to combine patients’ paired CT and steady-state metabolomics information to find a shared representation that can allow the reconstruction of both modalities. One benefit of using our two-step deep learning model is the ability to independently extract deep features from a single modality without needing another modality. This is of specific importance in the clinical setting, where it is often the case that a single data modality is more readily accessible than the other. Enhancing the features obtained from lesions on CT images, we are improving the usefulness of CT scans, which are more readily available when evaluating NSCLC patients for early diagnosis and tumor prognostication^33^.

In summary, we were able to show that there is a connection between the metabolomic and CT features of NSCLCs. Furthermore, it is possible to exploit the learned representation within CT images of patients with NSCLC that co-vary with tissue metabolomic profiles and demonstrate their usefulness clinically for histology subtype classification and prognosis on external datasets when only a CT scan is present.

## Methods

### Pre-processing of CT images

All image pre-processing was done using TorchIO, a package allowing for effective pre-processing of CT images^34^. To ensure comparability, the CT scans from all datasets were resampled to isotropic voxels of 1 × 1 × 1 mm. This was performed using linear and nearest neighbor interpolation for the image and segmentation, respectively^35^.

### Pre-processing data for training and testing DPCCA

We had 48 paired samples from the UHRS, each with two data views: CT scans and the metabolomic profiles of tumor/normal tissue. We performed a stratified split of the dataset into 32 paired samples for training, validation and 16 paired samples for testing whilst keeping the balance of AC/SCC consistent across the splits.

To ensure that the model was clinically relevant and matched the target population as much as possible we further considered the significance of the representative test data to ensure that our predictions hold clinical relevance. While assessing clinically-relevant predictions, we considered that the test data should match the target population rather than be a random subset of the same data pool as the train data^36^. To achieve this, we employed a multi-step stratification process that extended beyond the histological subtype: Split the dataset by histology, TNM8 Stage (due to clinical significance of staging), then gender (to account for potential variation in disease presentation between gender), and then age.

The stratification order was chosen based on its importance. We rationalized that ensuring the model could represent all stages and the frequency of occurrences (fewer females) was important, as such implemented this order in the test set. The following pre-processing steps were implemented on the training and testing data split separately.

Given the 3D segmentations, we calculated the center of mass (COM) and bounding box of the tumor. A 3D isotropic patch of 50 × 50 × 50, around the COM of tumor volume, was extracted, resulting in 48 3D tumor patches. We then created 3D patches of 32 × 32 × 32 randomly and ensuring that at least 65% of tumor was captured by the bounding box. The 3D patches were normalized to a range of 0–1 and lower upper boing of −1024 and 3021^35^.

For the metabolomics features, we only included those that were profiled in all patients for both tumor and non-tumor adjacent tissue, such that we had a total of 174 metabolites. The reason for this was to increase the reproducibility of the chosen features. Subsequently, we normalized the values of the metabolomic features to have a mean of 0 and a standard deviation of 1.

Data augmentation makes it possible to increase the data available for training without actually collecting new samples by applying a range of techniques. In this study, an augmentation factor of 186,624 was applied to the patches resulting in a training dataset of approximately nine million 3D patches. These augmentations were chosen based on other similar studies and consisted of ±18 pixels in three axes, random rotations at 90° intervals along the longitudinal axes, and random flipping along three axes^35^. The augmentations were applied in real-time during training, and simultaneously, we applied Gaussian noise with a standard deviation of 0.1 to the image patches and metabolomic features^35^. No augmentation was applied during validation or testing.

### Building DPCCA model

The DPCCA model is a deep generative model that fits the probabilistic canonical correlation analysis (PCCA) into two autoencoders, one for the CT image and the other for metabolomic. Figure 1 shows a detailed image of this model and where it fits the PCCA to the embeddings of two autoencoders. The code for this model was adapted from https://github.com/gwgundersen/dpcca. Specifically, we optimized the image autoencoder to enable studies with 3D images instead of 2D and used the 3D-DCGAN developed specifically for medical images^37,38^. The model was trained end-to-end using the mean squared error (MSE) for regression model fitting of paired CT image and metabolomics data; and also, for the reconstruction of the loss function for the modalities separately. The following section details the DPCCA method and its adaptation to our task.

Given a paired sample (**x**^a^, **x**^b^), the linear and convolution encoder embedded the CT images and metabolomics, respectively. These embedded vectors **y**^a^ and **y**^b^ are then fitted by the PCCA and incorporate an *l*_1_ penalty on the PCCA metabolomic weights, thus, encouraging sparsity in the metabolomic profiles and resulting in shared and view-specific latent variables **z** = [**z**^ab^ **z**^a^ **z**^b^]^T^.

Mathematically the PCCA can be expressed by Eq. 1 as follows:

1 $$\begin{array}{c}{z}^{{ab}} \sim N\left({O}_{k}{\rm{;}}{I}_{K}\right)\\ {z}^{a},{z}^{b} \sim N\left({O}_{k}{\rm{;}}{I}_{K}\right)\\ \begin{array}{c}{y}^{a} \sim N\left({\Lambda }^{a}{{\rm{z}}}^{{ab}}+{{\rm{B}}}^{a}{{\rm{z}}}^{a}{\rm{;}}{\Psi }^{a}\right)\\ {y}^{b} \sim N\left({\Lambda }^{b}{{\rm{z}}}^{{ab}}+{{\rm{B}}}^{b}{{\rm{z}}}^{b}{\rm{;}}{\Psi }^{b}\right)\end{array}\end{array}$$

Where ${B}^{j}\in {{\mathbb{R}}}^{{p}^{j}\times k}$, ${\Lambda }^{j}\in {{\mathbb{R}}}^{{p}^{j}\times k}$ and ${\Psi }^{j}\in {{\mathbb{R}}}^{{p}^{j}\times {p}^{j}}$. This can be reformulated as a factor analysis problem^18^, thus, suggesting that inference in the PCCA can be performed using expectation-maximization (EM), where the parameters are updated using the following tilling as seen in Eq. 2:

2 $$\begin{array}{c}{\Lambda }^{* }=\sum _{i}\left({y}_{i}{{\mathbb{E}}}_{z{{|}}{y}_{i}}{\left[z|{y}_{i}\right]}^{{\rm{{T}}}}\right){\left({{\mathbb{E}}}_{z{{|}}{y}_{i}}\left[z{z}^{{\rm{{T}}}}|{y}_{i}\right]\right)}^{-1}\\ {\Psi }^{* }=\sum _{i}\frac{1}{n}{diag}\left({y}_{i}{y}_{i}^{{\rm{{T}}}}-{\Lambda }^{*}{{\mathbb{E}}}_{z{{|}}{y}_{i}}{\left[z|{y}_{i}\right]{y}_{i}}^{{\rm{{T}}}}\right)\end{array}$$

Once the shared and view-specific latent variables $z={[{z}^{{ab}}{z}^{a}{z}^{b}]}^{{\rm{{\rm T}}}}$ are derived, the next step is to use the reparameterization trick to sample from the PCCA representation ${\hat{y}}^{j}{\mathscr{ \sim }}{\mathscr{N}}{\mathscr{(}}{\Lambda }^{{j}^{* }}{z}^{{ab}}+{{\rm{{\rm B}}}}^{{j}^{* }}{z}^{j};{\Psi }^{{j}^{* }})$ and obtain embedding samples ${\hat{y}}^{j}$. This step ensures that the Monte Carlo estimate of the expectation is distinct with respect to the encoder parameters and, thus, the model can be trained in an end-to-end fashion by defining the following loss function in Eq. 3:

3 $${\mathscr{L}}{\mathscr{=}}\frac{1}{n}\mathop{\sum }\limits_{i=1}^{n}\left({{\rm{||}}{\hat{x}}_{i}^{a}-{x}_{i}^{a}{\rm{||}}}_{2}^{2}+{{\rm{||}}{\hat{x}}_{i}^{b}-{x}_{i}^{b}{\rm{||}}}_{2}^{2}\right)+\gamma ({{\rm{||}}{\Lambda }^{b}{\rm{||}}}_{1}+{{\rm{||}}{\Lambda }^{{ab}}{\rm{||}}}_{1})$$

In the formulation described in this section, there are five hyperparameters (*p*^*a*^*,pb,k*^*ab*^*,k*^*a*^ and *k*^*b*^) determining the dimensions of the modality embeddings and latent space. In this case: $y\in {{\mathbb{R}}}^{p}$ such that *p* = *p*^*a*^ *+* *p*^*b*^, where *p*^*a*^ represents the dimensionality of CT embedding, and *p*^*b*^ represents the dimensionality of the metabolomics embedding. The latent space is $z\in {{\mathbb{R}}}^{k}$ where = *k*^*ab*^ *+* *k*^*a*^ *+* *k*^*b*^, $\Lambda \in {{\mathbb{R}}}^{p\times k}$ and $\Psi \in {{\mathbb{R}}}^{p\times p}$. To identify the best set of hyperparameters we did a grid search $p\in \{\mathrm{5,10,25,50}\}$ and k$\in \left\{\mathrm{2,3,5,10}\right\}$ such that *k* ≤ *p* was always satisfied and we selected the smallest number that resulted in a high image and metabolomics reconstruction. This was found to be *p*^*a*^ = *p*^*b*^ *=* 10 and *k*^*ab*^ *=* *k*^*a*^ *=* *k*^*b*^ = 3, such that *p* = 20 and *k* = 9, through the loss function defined in Eq. 3 and the reconstruction of both modalities as seen in Fig. 4.

### Building image autoencoder

The image autoencoder was based on the 3D-DCGAN similar to the image autoencoder in the DPCCA to make them directly comparable and trained using the mean squared error loss function to reconstruct the image. The same image preprocessing techniques described in “Pre-processing data for training and testing DPCCA” were used for this section and we performed image augmentation. A grid search was performed to identify the best hyperparameter, specifically the hyperparameter determining the size of the image embedding ${p}^{a}\in \{\mathrm{5,10,25,50}\}$. This was found to be *p*^*a*^ = 10, through the mean squared error loss function.

### Analysis of dataset for testing image embeddings for histology classification and survival

For this section, we trained our model on the TCIA cohort of (*n* = 203) and then performed the test on the test section of TCIA and three different datasets from the (*n* = 320) Royal Marsden Hospital (RMH), (*n* = 128) Guy’s and St Thomas’ Hospital (GSTT) and (*n* = 101) Imperial College Healthcare Trust (ICHT)^25^. We first filtered the datasets only to have patients with AC and SCC histology.

As a baseline for feature quality, we used TextLab 2.0 software to extract 438 features from the lesion. The methods in Table 6 were applied to features extracted using the DPCCA, image autoencoder and TextLab 2.0, the latter for radiomics analysis.

**Table 6:** Summary of the feature selection and prediction methods used, if (*) method is used for classification, (**) used for survival, otherwise method is used for both classification and survival.

Acronym	Feature selection methods	Acronym	Prediction methods
VAR*	Variance	GNB*	Gaussian naïve baye
RELF*	Relief	MNB*	MultinomiIive bayes
MI*	Mutual information	BNB*	Bernoulli naïve bayes
mRMR*	Minimum redundancy maximum relevance ensemble	KNN*	K-nearest neighbourhood
ETE*	Extra tree ensemble	RF*	Random forest
GBDT*	Gradient boosting decision tree	BAG*	Bagging
TSQ*	T-test score	DT*	Decision tree
CHSQ*	Chi-square score	GBDT*	Gradient boosting decision tree
EN*	Elastic net	Adaboost*	Adaptive boosting
LASSO*	Least absolute shrinkage and selection operator	XGB*	Xgboost
WLCX*	Wilcoxon	LDA*	Linear discriminant analysis
L^1^-SVM*	L^1^- based linear support vector machine	LGR*	Logistic regression
L^1^-LGR*	L^1^ -based logistic regression	Linear-SVM*	Linear support vector machine
JMIM**	Joint mutual information maximisation	RBF-SVM*	Radial basis function support vector machine
RFVH**	Random forest with variable hunting	MLPC*	Multi-layer perceptron
RFVH -IMP**	Random forest with variable hunting and Gini impurity corrected variable importance	BGLM_CoxPH**	Boosting gradient linear models
RF*	Random forest variable hunting with maximal depth	BGLM_Cindex**	Boosting gradient linear models
Spearman**	Spearman correlation	BGLM_Weibull**	Boosting gradient linear models
Person**	Pearson correlation	BT_CoxPH**	Boosting trees
Kendall**	Kendall rank correlation	BT_Weibull**	Boosting trees
Random**	Random (null hypothesis)	Cox_Lasso**	Cox lasso
		Cox_Net**	Cox net
		Cox**	Cox proportional hazard
		RSF**	Random survival forest
		MSR_RF**	Random forest using maximally selected rank statistics
		ET_RF**	Random forest with extra trees

There exists a wide range of feature selection and machine learning techniques. Identifying the feature selection and machine learning algorithm is task-dependent and critical when developing clinically applicable models. Therefore, we combined different feature reduction techniques for the classification and survival tasks^39^. To find the best combination, we performed a ten-fold cross-validation using the training split of the TCIA data. Then, we used the average accuracy to select the best feature reduction and machine learning algorithms. The acronyms of each feature section, classification and survival method are defined in Table 6.

For the histology classification task on radiomic features, we selected 15 feature selection methods and combined them with 12 machine-learning classifiers based on previous related research^39,40^. The filter selection methods consisted of univariate and multivariate filter methods, which are classifier-independent and embedded methods such as penalty and tree-based methods, which incorporated the feature selection in the training process. For the classification task, we selected a broad range of methods as suggested by previous studies. We used a cross-combination strategy to select the method with the best mean F1 score across the ten-fold validation^41^. The feature selection and classification task were performed using the scikit-learn package in Python^42^.

In the survival analysis, we used a different set of feature selection methods and models as suggested by the literature and included the Cox proportional hazard model as a benchmark^43^. These specifics are capable of handling censored, heterogenous and high-dimensional data. The machine learning algorithms selected in this section can be divided into four categories: penalized Cox regression, boosted Cox regression (GLM), boosted based on trees and random forests. To select the best combination, we used a cross combination strategy to select the method with the best mean C-index across the ten-fold validation. The feature selection and classification task were performed using the ‘survival’ package in R^44^.

The best feature selection and machine learning model combination were then selected to perform histology subtype classification and survival analysis using the TCIA validation cohort and the three external datasets, as reported in Tables 3 and 4.

### Ethics

This study used retrospective human data and complied with all relevant ethical regulation except where this was waived. Specifically three types of retrospective data were used: (a) Dataset obtained from the University Hospital Reina Sofia (UHRS), Spain. The research study was conducted in accordance with the Helsinki Declaration and was approved by the Cordoba Clinical Research Ethics Committee; all patients provided a signed written informed consent for participation in the study. In addition to CT scans. Correlated tissue Tissue samples were stored by the Andalusian Health Services Biobank, and the metabolomic profiling was performed under contract by Metabolon as reported previously^19^. (b) OCTAPUS-AI dataset (UK). OCTAPUS-AI represents a study from multiple UK cancer centers including Guy’s and St Thomas’ NHS Foundation Trust, Imperial College Healthcare NHS Trust and the Royal Marsden NHS Foundation Trust, collected for the explicit purpose of developing robust predictive lung cancer algorithms as previously reported^25^. This study was approved by the UK Health Research Authority (reference number: 20/HRA/3051); ClinicalTrials.gov identifier, NCT04721444. As the data used in the study were de-identified, patient consent was not required for this type of study and as per the respective Health Research Authority and Research Ethics Council approvals. (c) TCIA dataset. The Cancer Imaging Archive (TCIA) provides the cancer research community with an open-source repository of de-identified and highly curated radiology and histopathology imaging data (www.cancerimagingarchive.net). In keeping with TCIA’s grant-funded mandate from United States National Institute of Health, the dataset is considered de-identified information as defined by the Health Insurance Portability and Accountability Act of 1996, as amended (“HIPAA”). Institutional Review Board approval for TCIA data was not required for use of the dataset.

### Reporting summary

Further information on research design is available in the Nature Research Reporting Summary linked to this article.

### Supplementary information

Reporting Summary

Supplementary Infprmation

## Data Availability

The TCIA data is publicly available from https://wiki.cancerimagingarchive.net/display/Public/NSCLC-Radiomics. The UHRS dataset is not publicly available but can be request to the corresponding authors. The (GSTT, Imperial and RMH). data are not publicly available but can be requested to the corresponding authors and/or OCTAPUS-AI.

## Code availability

The underlying code used to reproduce the key findings are publicly accessible in Mendeley Data with the identifier 10.17632/ft2f3xhjz7.1^38^.
