# Supplementary material for: Deep representation learning of tissue metabolome and computed tomography annotates NSCLC classification and prognosis
Source: NPJ Precis Oncol. 2024 Feb 3;8:28. doi: 10.1038/s41698-024-00502-3 (PMC10838282; doi:10.1038/s41698-024-00502-3)
Supplement: Supplementary file 2 — Supplementary Infprmation [file 41698_2024_502_MOESM2_ESM.pdf]

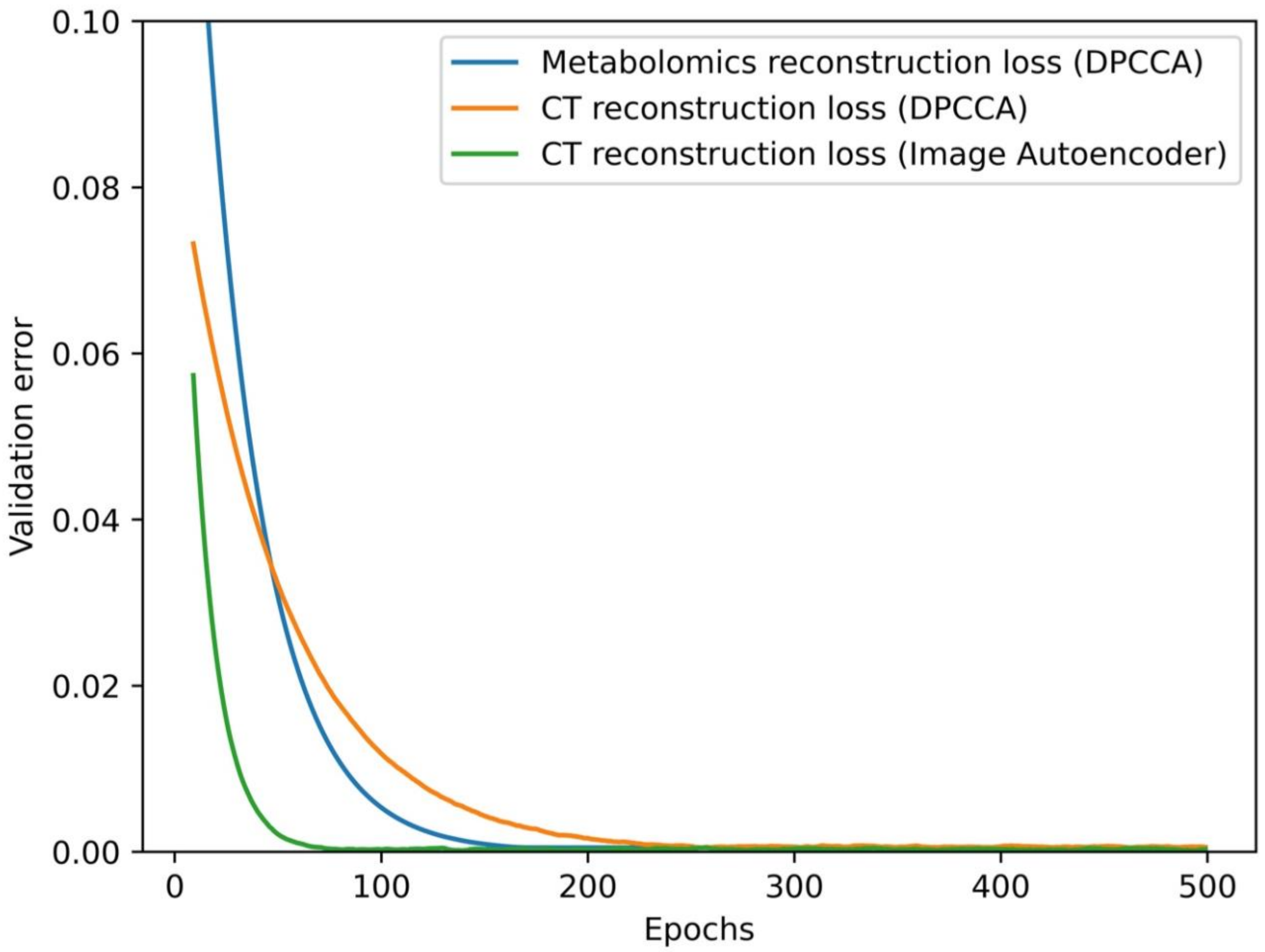

Supplementary Figure 1. Validation error of DPCCA for each view over training on validation data from UHRS and for image autoencoder on the CT view.

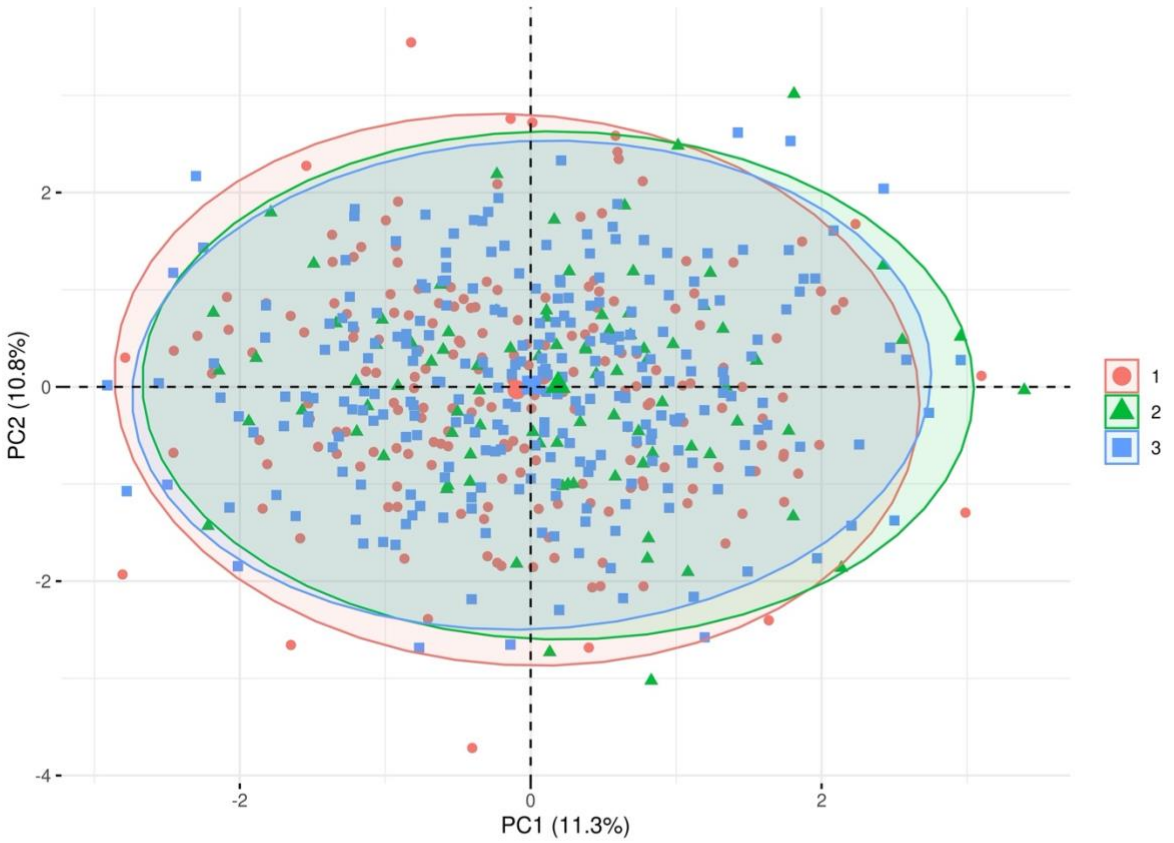

Supplementary Figure 2. Principal component analysis (explained variance) of TMR-CT to assess congruence of data from different TNM8 overall stage of nodules: stage 1, stage 2 and stage 3.

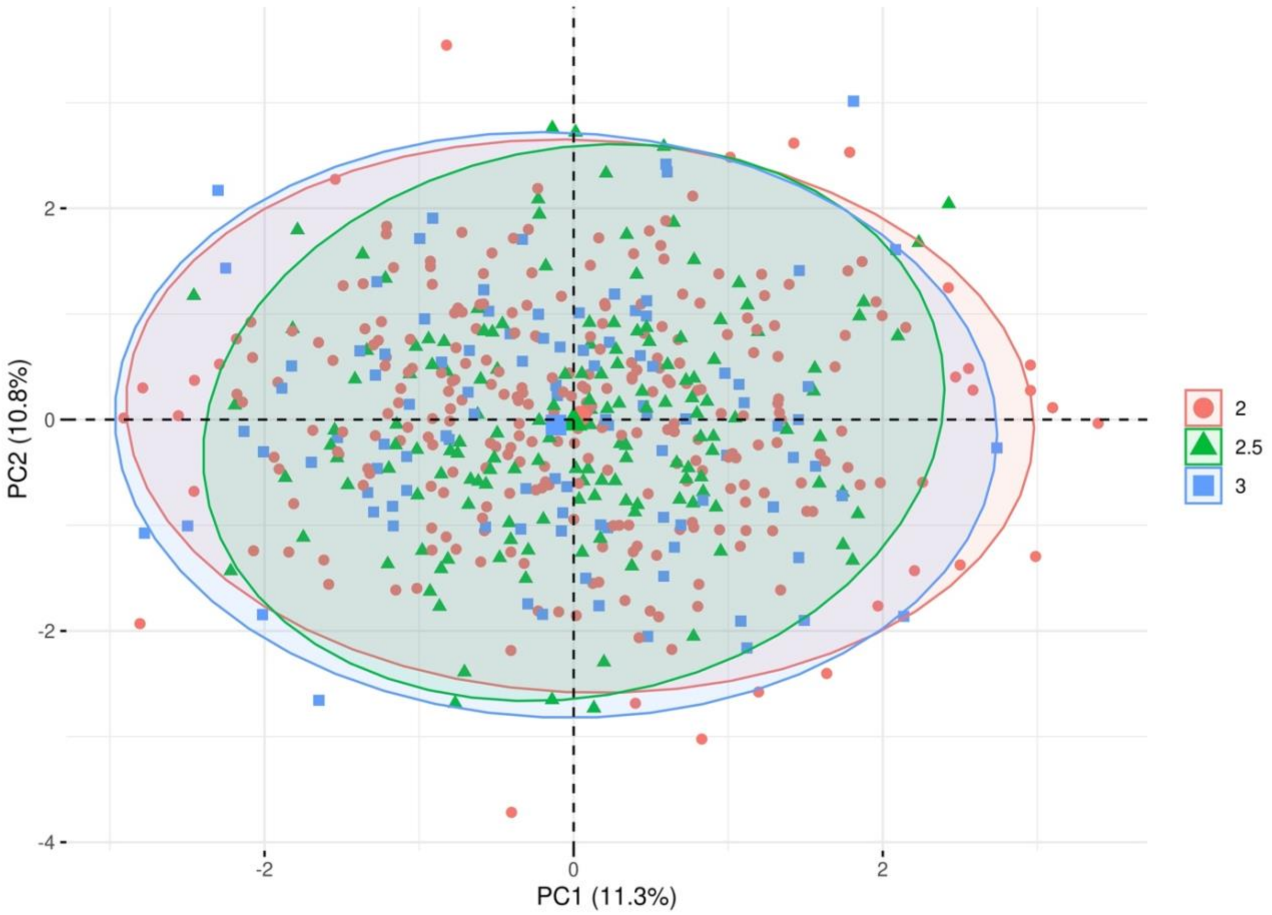

Supplementary Figure 3. Principal component analysis (explained variance) of TMR-CT to assess congruence of data from different thickness of CT thickness: 2, 2.5 and 3 mm.

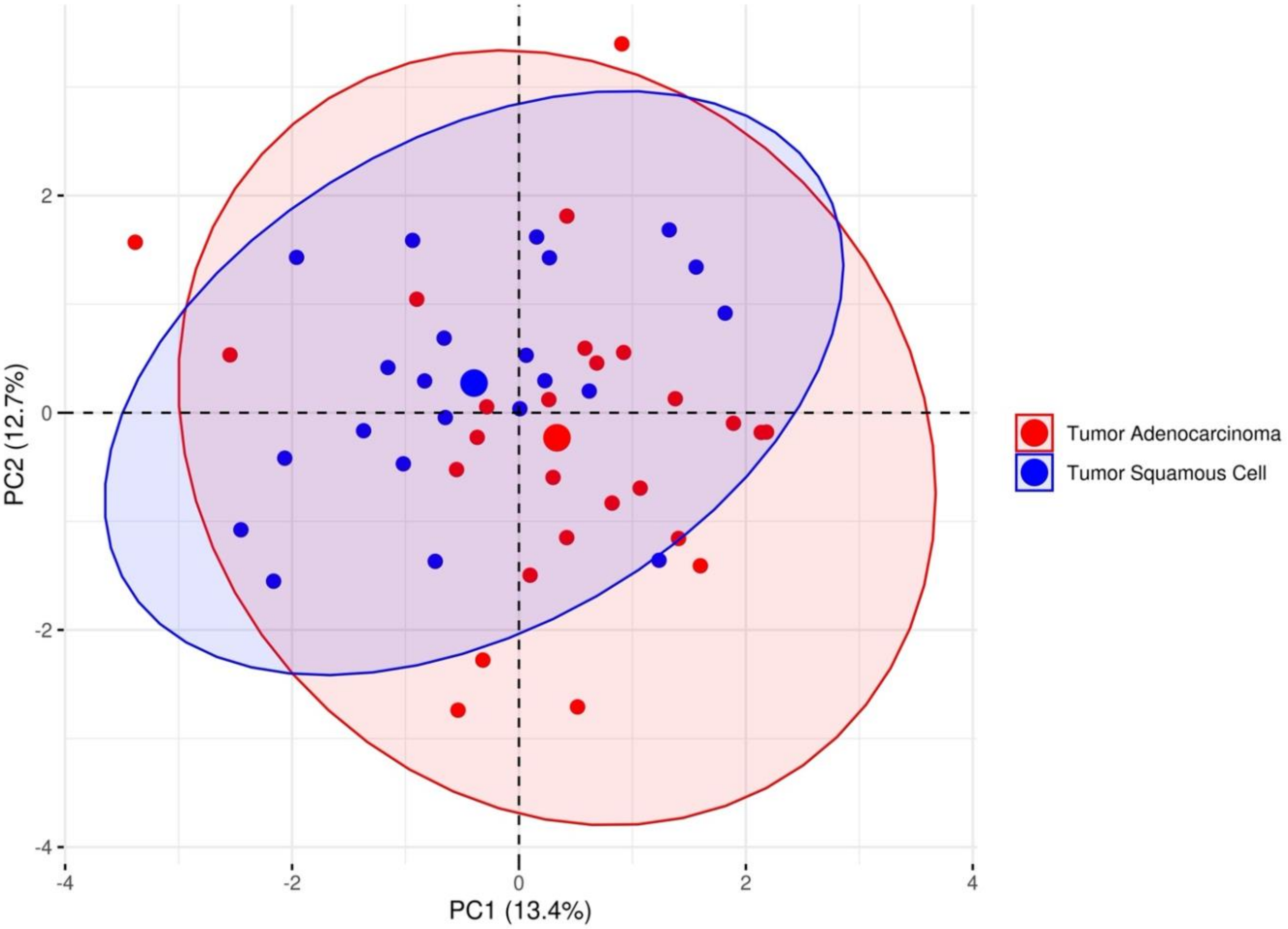

Supplementary Figure 4. Principal component analysis (explained variance) of CT_emb features from UHRS dataset for distinguishing between AC and SCC.

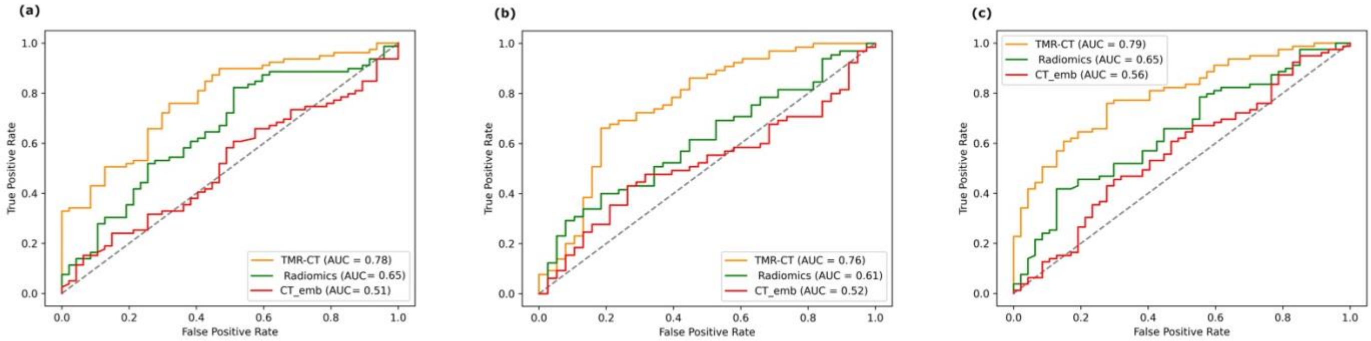

Supplementary Figure 5. ROC curve for classification of AC and SCC using TMR-CT, radiomics and CT_emb features on (a) GSTT, (b) ICHT and (c) RMH. The area under the curve (AUC) is shown in the legend for each plot.

OCTAPUS-AI Consortium Members:
Sumeet Hindocha[1], Thomas G. Charlton[2], Benjamin Hunter[1,3,4], Charleen Chan[3], Merina Ahmed[5], Matthew Orton[6], Jason Lunn[3], Simon J Doran[3], Shahreen Ahmad[2], Fiona McDonald[1,3], Imogen Locke[5], Danielle Power[7], Matthew Blackledge[8], Richard Lee[1,9]

[1]Early Diagnosis and Detection Centre, National Institute for Health and Care Research Biomedical Research Centre at the Royal Marsden and Institute of Cancer Research, London, SW3 6JJ, United Kingdom. [2]Guy's Cancer Centre, Guy's and St Thomas' NHS Foundation Trust, Great Maze Pond, London, SE19RT, UK. [3]Institute of Cancer Research NIHR Biomedical Research Centre, London, UK. [4]Cancer Imaging Centre, Department of Surgery & Cancer, Imperial College London, Du Cane Road, London, W12 0NN, UK. [5]Lung Unit, The Royal Marsden NHS Foundation Trust, Downs Road, Sutton, SM25PT, UK. [6]Artificial Intelligence Imaging Hub, Royal Marsden NHS Foundation Trust, Downs Road, Sutton, SM25PT, UK. [7]Department of Clinical Oncology, Charing Cross Hospital, Fulham Palace Road, London, W6 8RF, UK. [8]Radiotherapy and Imaging, Institute of Cancer Research, 123 Old Brompton Road, London, SW7 3RP, UK. [9]National Heart and Lung Institute, Imperial College London, Guy Scadding Building, Dovehouse Street, London, SW3 6LY, United Kingdom.
